# ArEEG: an Open-Access Arabic Inner Speech EEG Dataset

**DOI:** 10.1038/s41597-025-05387-w

**Published:** 2025-08-29

**Authors:** Donia Metwalli, Antony E. Kiroles, Yousef A. Radwan, Eslam Ahmed Mohamed, Mariam Barakat, Anas Ahmed, Amr M. Omar, Sahar Selim

**Affiliations:** 1 Present Address: Center for Informatics Science (CIS), School of Information Technology and Computer Science, Nile University, 26th of July Corridor, Sheikh Zayed City, Giza, 12588 Egypt; 2 School of Engineering, Nile University, 26th of July Corridor, Sheikh Zayed City, Giza, 12588 Egypt

**Keywords:** Cognitive control, Cognitive neuroscience

## Abstract

Recent advancements in Brain-Computer Interface (BCI) technology are shifting towards inner speech over motor imagery due to its intuitive nature and broader command spectrum, enhancing interaction with electronic devices. However, the reliance on a large number of electrodes in available datasets complicates the development of cost-effective BCIs. Additionally, the lack of publicly available datasets hinder the development of this technology. To address this, we introduce a new Arabic Inner Speech dataset, featuring five distinct classes, exceeding the typical four-class datasets, and recorded using only eight electrodes, making it an economical solution. Our primary objective is to provide an open-access, multi-class Electroencephalographic (EEG) dataset in Arabic for inner speech, encompassing five commands. This dataset is designed to enhance our understanding of brain activity, facilitate the integration of BCI technologies in Arabic-speaking regions, and serve as a valuable resource for developing and testing real-world BCI applications. Through this contribution, we aim to bridge the gap between language-specific neural data and the field of neurotechnology, fostering innovation and inclusivity in BCI research.

## Background & Summary

Brain-Computer Interface (BCI) is a computer-based system that utilizes electric neuron activity within the brain to create a direct communication channel between the brain and a computer, with the purpose of transmitting messages and commands in real-time^1–3^. Hence, BCI is considered a breakthrough in the field, as it paves the way for easier communication methods for individuals who struggle with communication disabilities^4,5^ caused by different disorders such as locked-in syndrome^6,7^ and aphasia^8^, which affects their day-to-day lives. BCI systems are generally classified as either non-invasive or invasive. To emphasize, invasive techniques provide highly accurate signals but are expensive and necessitate brain surgery, hindering their ease of use. On the other hand, non-invasive methods involve placing sensors on the scalp without the need for surgery, offering a more cost-effective, efficient, and safe solution for various BCI applications. However, the quality of the signals acquired may be lower due to factors such as signal attenuation through the skull, susceptibility to noise and artifacts, as well as reduced spatial resolution compared to invasive methods^3,9–11^. The most common recording modalities utilized by BCI systems include electroencephalographic (EEG) signals as well as alternative methods like magnetoencephalography (MEG) and electrocorticography (ECoG)^12,13^. Among the prevalent paradigms in EEG-based BCI systems are P300, which is acquired as a result of an external stimulus, and Steady-State Visually Evoked Potentials (SSVEP), which is produced as a response to repeatedly flashing lights^5,13^.

While these paradigms have notably improved EEG-based BCI systems, they may not always be optimal for device control in specific scenarios. This is mainly attributed to slow response times or the significant user effort involved, as well as their dependence on visual and external stimulants, thereby restricting their feasibility for real-world and prolonged use. Paradigms such as motor imagery^14^ and inner speech^15^ address these limitations effectively. Motor imagery can be described as the brain activity that is activated when one envisions or mentally simulates the movement of various body parts and limbs^14,16^. Inner speech involves the internalized process where individuals engage in thinking based on pure meanings, often accompanied by an auditory representation of their inner voice. This experience is also recognized as verbal thinking or inner speaking^7,15^. Motor imagery relies on physical movement, making its signals relatively easy to identify^14,16^. In contrast, while inner speech poses greater challenges due to its inaccuracy, it excels in overcoming some of the constraints present in different types of EEG signals. For instance, inner speech is not restricted to physical movement as motor imagery. Additionally, inner speech does not need any visual stimulant, such as P300 signals. However, due to the novelty of the technology, the number of publicly available inner speech EEG datasets is very limited, which hinders its further advancements. Furthermore, the most common number of classes present in available inner speech datasets is 4, such as^15^. Furthermore, since these signals are precise and can be difficult to acquire, a headset with a significant number of electrodes, i.e. 128 electrodes, should be used to guarantee good signal acquisition. Unfortunately, such headset can be highly expensive and not accessible.

In this study, we introduce a publicly available dataset which includes 5 Arabic classes and utilizes non-invasive inner speech EEG signals, contributing to multiple aspects such as; an increased number of classes and addressing the absence of existing Arabic datasets. Furthermore, the data was collected in a cost-effective manner by using an 8-channel headset. Introducing a dataset of this nature will have a significant impact on the expansion of research in the MENA region and Arabic-speaking countries, enabling them to utilize inner speech EEG-based BCI technology. Additionally, it will allow non-Arabic speaking countries to further investigate the potential of this technology and use the dataset as a foundation for advancements in the classification of inner speech signals. By providing a robust and comprehensive dataset, researchers will be better equipped to explore and refine techniques in this emerging field, ultimately bringing the science of inner speech signals closer to achieving its full potential.

In the subsequent sections of the paper, we delve into various aspects of data collection and validation. To elaborate, the Methods section furnishes an outline of the experimental design and the procedures involved in acquiring data from participants, including details on hardware, software tools, and the experimental process. Transitioning to the data records section, a comprehensive description is provided of the raw recorded data, preprocessed data, the significance of technical data validation, feature engineering, different methodologies employed, and the utilization of machine learning and deep learning classifiers. The paper wraps up by summarizing the research findings and the contributions made in the study.

## Methods

This section provides a detailed description of the methods used to conduct the experiments. An overview of the participants’ background is shared, as well as the experiment software, setup, and procedures followed to get a clearer view of how the dataset was collected.

It is important to note that the experimental procedure was proposed to and approved by the ITCS Ethics Committee of Nile University in Egypt. They authorized the conduction of the experiments and the study, as well as using the data collected. The relevant case number for this study is ITCS-EAN-1-4-2024.

### Participants

The dataset comprises inner speech EEG signals from 12 healthy adult participants, seven males and five females, aged 17 to 25. All are native Arabic speakers and fluent in English, facilitating accurate data collection. Before the experiment was conducted, the process of data collection was explained in detail to the participants to ensure they were aware of the process before consenting. All participants selected signed a consent form before the commencement of the experiments. The consent form explicitly stated that their brain activity could be recorded and used for research purposes, including publication as a publicly available dataset for unrestricted use by other researchers.

The participants consented to open sharing of their anonymized EEG data. Hence, to maintain their privacy, all personal identifiers were removed. To elaborate, only the age and gender were retained, while names, any contact information, and demographic details were omitted. The reasoning behind the inclusion of the aforementioned information is to help in assessing the dataset and ensure the collected signals are representative of a specific age group, avoiding significant age-related variability. Additionally, including the gender of each participant allows one to verify that the dataset is balanced in terms of gender representation.

Each participant committed to approximately 100 minutes of recordings (excluding rest time between recordings), distributed across 15 sessions, averaging seven minutes each. It should be noted that one of the 12 participants selected completed a total of 21 sessions, rather than the initially selected 15 sessions. This was done to analyze and evaluate how the classification results, which will be mentioned in the upcoming sections, will be affected with increasing the data per subject. Accordingly, with the results not changing significantly, the initially chosen number of sessions was maintained. Notably, none of the participants had prior experience with BCI experiments, necessitating detailed explanations of the process. Detailed participant information can be found in the Table 1

**Table 1:** Participants information.

Subject	Gender	Age	Native Language	Dominance
Subject 0	Male	21	Arabic	Right
Subject 1	Male	25	Arabic	Right
Subject 2	Male	21	Arabic	Right
Subject 3	Male	22	Arabic	Right
Subject 4	Male	21	Arabic	Right
Subject 5	Female	21	Arabic	Right
Subject 6	Male	23	Arabic	Right
Subject 7	Female	22	Arabic	Right
Subject 8	Female	17	Arabic	Right
Subject 9	Female	19	Arabic	Right
Subject 10	Female	19	Arabic	Right
Subject 11	Male	21	Arabic	Right

### Hardware

**Fig. 1:**
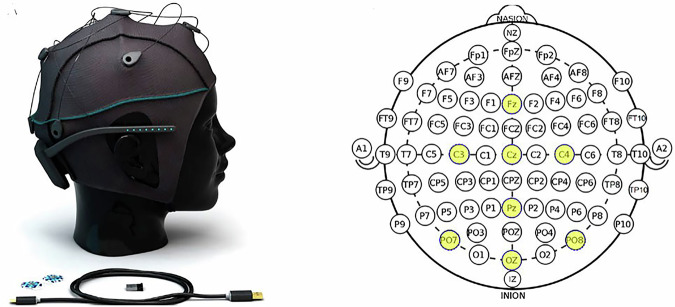
Unicorn Hybrid Black Dataset and Electrode Placement. The figure shows the 8-channel electrode configuration used in the study, following the international 10–20 system. Electrode positions include FZ, C3, CZ, C4, PZ, PO7, OZ, and PO8. This setup balances spatial resolution with affordability and accessibility^18^.

The experiment was done using g.tec’s Unicorn Hybrid Black + headset 1, which is an 8-channel (i.e., 8-electrode) headset running at a 250 Hz sampling rate. Using an 8-channel headset ensures the accessibility and affordability of the EEG technology. However, these advantages are guaranteed at the expense of the spatial resolution of the signal acquired. To elaborate, utilizing high-density headsets, i.e. headsets with more than 32 channels, allows us to record brain activity from many locations across the scalp. Doing so increases the spatial resolution of the signal acquired, as the system can then detect where the signals acquired originate more precisely. While the aforementioned point is critical to ensure the accuracy and precision of any EEG system, this paper chose to utilize an 8-channel system for practicality — particularly in contexts where cost, portability, and ease of use are essential. Recent research^17^ indicates that a number of signal processing and feature extraction techniques can be done on the low-resolution signals acquired to enhance their quality. Accordingly, this research depended heavily on this factor. To elaborate, a number of different preprocessing and feature extraction techniques have been implemented and utilized to find the most suitable technique to enhance the signal’s quality as much as possible. In doing so, the confidence in the reliability of the acquired signals is increased. Additionally, it proposes a demonstration that accurate inner speech classification can indeed be achieved with affordable, low-density EEG headsets. The data was collected using the electrodes placed at FZ, C3, CZ, C4, PZ, PO7, OZ, and PO8 positions according to the 10–20 system, as can be seen in Fig. 1. The headset was provided with caps that are configured with the electrodes placed at the aforementioned positions. As an extra measurement to ensure that the signals collected were accurate and reliable, two caps of different sizes, medium and large, were used depending on the participant’s head circumference. This guarantees that the electrodes were placed correctly and were not loose.

### Experiment software

The data collection software is a Python program running on top of the Unicorn Linux Software Development Kit (SDK)^18^, which allows us to connect the headset to the Python program that displays the commands and collects the desired data. As such, the software was run on Ubuntu 20.04 Long Term Support (LTS). Additionally, it utilizes OpenCV^19^ to show the visual cues and PyGame^20^ to play the audio cues. Details on these cues will be provided in a later section. The software is also designed to automatically annotate the recorded words as they are shown to the participants, ensuring the accuracy of labels and timestamps and preventing data mislabeling.

The visual cues in the data collection software were displayed in white font on a black background to minimize distractions and simplify the interface, enhancing focus for the participants. The text for the visual cues was categorized into two types: the “Rest” instruction was written in English, and the class names that were needed to be recorded were written in Egyptian Arabic. The “Rest” instruction was displayed in English to be as distinct as possible from the classes so as to avoid the confusion of thinking that they need to perform the inner speech task for the “Rest” instruction. The audio cue is a beep sound from Pixabay^21^, a free audio-sharing platform, that plays when the participant is asked to press a button to move to the next visual cue. The duration between the start of the audio cue and pressing a button and the following cue is marked in the recorded data with a separate tag, which is “Wait” to avoid tainting the classes with any irrelevant signals

### Experimental procedure

Upon entering the room designated for conducting the experiments, the experimental process was explained to the participants. Furthermore, the EEG headset and reference electrodes were placed and verified for correct positioning according to the process in the Unicorn Hybrid Black + manual^18^. The participants were then seated in front of a laptop that is connected to the headset via Bluetooth and ran the data collection software on Linux. The setup process took approximately 20 minutes, with an additional 20–30 minutes to explain the experiment procedure to the participant and an additional test run of the recording session to familiarize the participant with the experiment. After the setup and briefing, the room is blacked out with no sources of light other than the laptop screen to reduce distractions and minimize the noise in the signal. Each participant recorded 15 sessions split over 2 to 3 days of recording to prevent fatigue and maintain signal quality. Each session consists of 25 trials, where each trial consists of 15 seconds: 10 seconds of rest followed by 5 seconds of the target class. A 30-second buffer was added at the start of each session, as recommended by the EEG headset manufacturer, to enhance signal quality and stability. As such, each session adds up to 405 seconds, or roughly 7 minutes per session. Participants were also allowed to take breaks between sessions as needed. With the sessions divided across multiple days, it was crucial to ensure the environment, as well as the participant’s mental state, was the same throughout all sessions. This aims to maintain consistency and minimize external factors that could influence the results. To emphasize, EEG signals can vary widely based on the mental state of the participant and the surrounding environment^22^. For instance, the signals may be slightly weaker or different if the participant is not well rested or stressed, or include noise if the surrounding environment is distracting. Hence, to minimize the impact of external factors as much as possible, all experiments were held in the same room and same place, with the temperature maintained and with minimal surrounding external noise. Additionally, the participants were always asked before the scheduled sessions regarding their mental state and health condition. In doing so, it was ensured that the experiments were conducted in similar conditions, where the participant slept an approximately equal amount of hours beforehand and was dealing with minimal stress and had no new health issues. If the aforementioned conditions were not as required, the sessions were postponed to a more suitable time. It is important to note that along with maintaining the external factors during the sessions, the electrode placements remained the same for all participants. Furthermore, certain steps were conducted between sessions each day to ensure the signals included minimal noise and were accurate. These steps included changing the reference electrodes, placed behind both ears, every 3 sessions. Furthermore, participants were allowed to take breaks upon request, as previously mentioned. Their alertness was assessed every two sessions through self-tests and reaction-time tests to ensure they maintained focus. The classes were selected to align with the standard classes provided in other inner speech EEG datasets, such as^15^, which were chosen since they fit with the notion of a control application,^15^. However, since the aim is to increase the number of classes, an additional class was added, which is “Select”. As such, the words used were the Arabic equivalent of “up,” “down,” “left,” “right,” and “select”. This focus on control-relevant vocabulary ensures that our research transcends mere linguistic decoding and holds tangible potential for developing assistive technologies and communication interfaces tailored for Arabic speakers. These words were chosen because they provide a clear, standardized framework for classification, making it easier to analyze patterns and compare results across studies. Additionally, directional terms offer an intuitive and consistent way to structure the dataset, ensuring meaningful evaluation and validation of the classification model, as well as the dataset itself. It is important to note that although each class is presented an equal number of times for every participant, the order in which the classes appear is randomized and differs across trials. Due to the separation of sessions across multiple days, all participants were able to record the same number of trials across all sessions, with each participant recording 375 trials, except for subject 3 with 525 trials. As a result, the experiments resulted in the collection of 4650 trials in total.

**Fig. 2:**
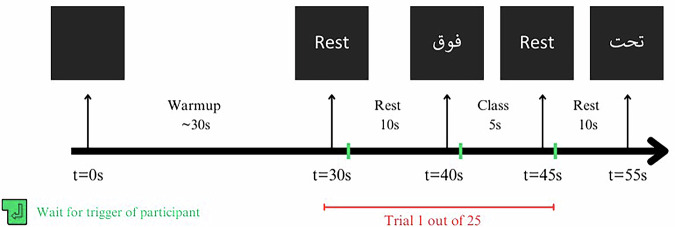
Timing diagram of a single session trial. Each trial spans 15 seconds, consisting of 10 seconds of rest and 5 seconds of inner speech of one class shown on the screen. The diagram highlights key timestamps: the visual cue presentation, the participant’s response, where they need to press a key to change the visual cue from’Rest’ to a class, and resting phases. The session begins with a 30-second warm-up period for signal stabilization.

As shown in Fig. 2, each session begins with a warm-up duration of 30 seconds during which the participants are instructed to close their eyes and clear their thoughts, which is necessary to stabilize the signals. At t = 30 s, an audio cue is played, marking the beginning of the first trial of 25 trials. The audio cue is then followed by the first trial, which starts with 10 seconds of resting, where the participants are again instructed to close their eyes and enter a resting state with little to no thoughts. At t = 40 s, the audio cue plays, which prompts the participants to open their eyes, press any button on the keyboard to show the class visual cue, read the cue, and close their eyes again. The visual cue stays on the screen for 5 seconds, during which the participant is instructed to imagine the words as if they are saying them in their internal monologue voice repeatedly, resulting in the desired inner speech signals. At t = 45 s, an audio cue is played, signaling to the participant to stop thinking of the class, open their eyes, press any button on the keyboard, close their eyes, and enter a rest state, which is the start of the next session. The trial sequence is repeated for the remaining 24 trials with the same sequence from t = 30 s to t = 45 s without any external intervention or breaks. At the end of the session, the participant is asked whether or not they want to take a break before the next session commences.

### BCI interaction conditions

The design of the dataset and experiment aimed to capture, decode, and understand EEG signals of inner speech with short, clear breaks between trials and fewer electrodes than previous research, such as that of^15^. During the experiment, participants were instructed to close their eyes and minimize cognitive activity during the “Rest” commands to ensure clean signal capture. For each trial, upon the display of a visual cue corresponding to one of the five predefined classes, participants were asked to internally verbalize the prompted word using their inner monologue voice, while remaining as still as possible to avoid introducing noise into the EEG data. The audio cue was provided to signal an event change.

### Data processing

Data processing and transformation were implemented using NumPy^23^ and Pandas^24,25^ in Python to ensure data consistency and clarity of the recorded data.

The recorded signal, extracted from the csv file generated from recording, was loaded into a flat matrix of shape [*t* × 8], where t is the number of recorded samples. It was then converted to a matrix of shape [*n* × 8 × 1200], where n is the number of trials and each row represents an 8-channel segment of data points grouped by state or trial. This restructuring facilitated easier segmentation and analysis of EEG data, organizing it into cohesive segments based on experimental states. Afterwards, to enhance data consistency, the number of data points for all classes was standardized to 1200, previously ranging from 1200 to 1250. This involved removing extra classes (“Rest”, “Wait”, and “Warm Up”), leaving the data to include solely the five collected classes.

**Fig. 3:**
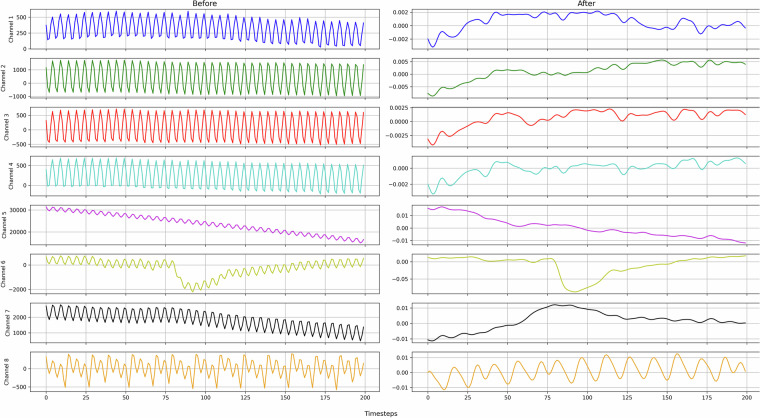
EEG signal before and after preprocessing. This plot compares a raw EEG segment with its filtered version after applying a notch filter at 50 Hz and a band-pass filter (0.5–30 Hz). The preprocessing effectively reduces power-line noise and enhances signal clarity for subsequent analysis.

Moreover, to enhance data quality, several signal processing techniques were applied, starting with a notch filter with a frequency of 50 Hz to eliminate power-line interference. Additionally, a band-pass filter ranging from 0.5–30 Hz with a sampling frequency of 250 Hz was implemented to focus on relevant frequency components. Each trial data was processed using these filters, and the resulting signals were scaled to ±50 µV for consistency and comparability across trials. Figure 3 illustrates the data from a 200-timestep segment of a single trial before and after processing, highlighting the effects of the filtering and scaling techniques applied.

Additionally, the code, raw data, and transformed data are freely available for users to adapt and utilize according to their specific needs and preferences. For more details on accessing these resources, please refer to the Code Availability section.

## Data Records

**Fig. 4:**
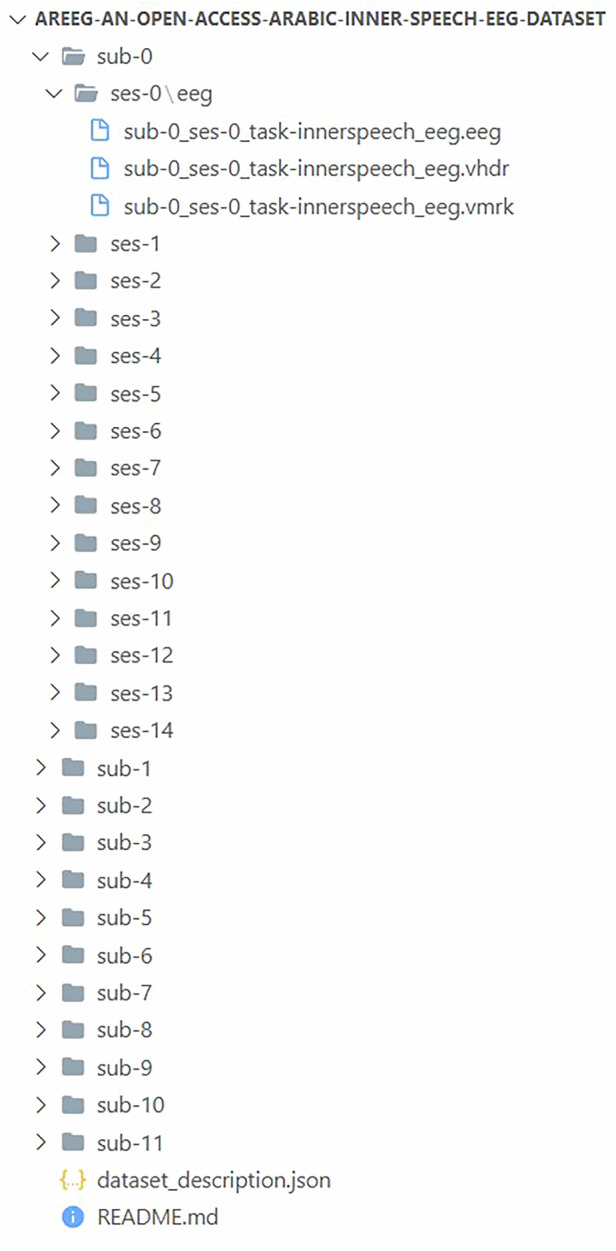
Hierarchical structure of the dataset in the OpenNeuro repository. The structure includes folders for each of the 12 participants. Each of these contains subfolders corresponding to individual sessions, where the raw data files are stored. One participant (subject 3) includes 21 sessions for the reasons aforementioned, while the rest contain 15 sessions each.

The dataset is publicly available and can be found in OpenNeuro Repository^26^. As previously mentioned, the experiments were carried out on 12 different participants, with each participant recording 15 sessions. The main folder named “Arabic Inner Speech EEG Dataset” includes a subfolder titled “Raw Data”. The subfolder contains 12 folders, where each participant has a respective folder that stores all 15 sessions except for subject 3 with 21 sessions for the previously mentioned reason. To clarify, the hierarchy of the folders can be seen in Fig. 4. In addition to the raw data, the preprocessing steps and code utilized are also provided. This setup allows researchers to freely process the raw data as described in this study and modify it according to their specific research needs.

### Raw data

The raw data files encompass all readings recorded during the sessions. These files are organized into eight classes: five main classes previously described and three additional classes—“Warm Up”, “Wait”, and “Rest”. Due to variations in the data collection process and participant response rates, the number of readings for each class varies. The five main classes contain between 1200 and 1250 readings per class. Since each class is repeated five times per session, the total number of readings for each class ranges from 6000 to 6250 in a single session. The “Rest” class contains up to 2500 readings between each word. The “Wait” class readings are the most variable, largely depending on the response rate of each participant. The signal frequency for all classes is set at 250 Hz.

### File formats

All the files in the OpenNeuro repository^26^ follow the BIDS specification^27^ format. To access the data, the dataset needs to be imported into Python and can be accessed and saved using the MNE library^28^. The files are uploaded in the Brain Vision Core format as mentioned in^27^. To load the data use the “mne.io.read_raw_brainvision” function in MNE library, or any other library that supports reading the Brain Vision Core format.

## Technical Validation

All visualizations in this section are based on the recorded segments of the five main classes, along with one second preceding each segment, which falls under the “Wait” class. This is done to capture the transition between the activity before the visual cue and during it. The duration of each trial was fixed to 1200 timesteps and all subjects’ data was used as will be seen in the following visualizations.

### Modeling performance

**Fig. 5:**
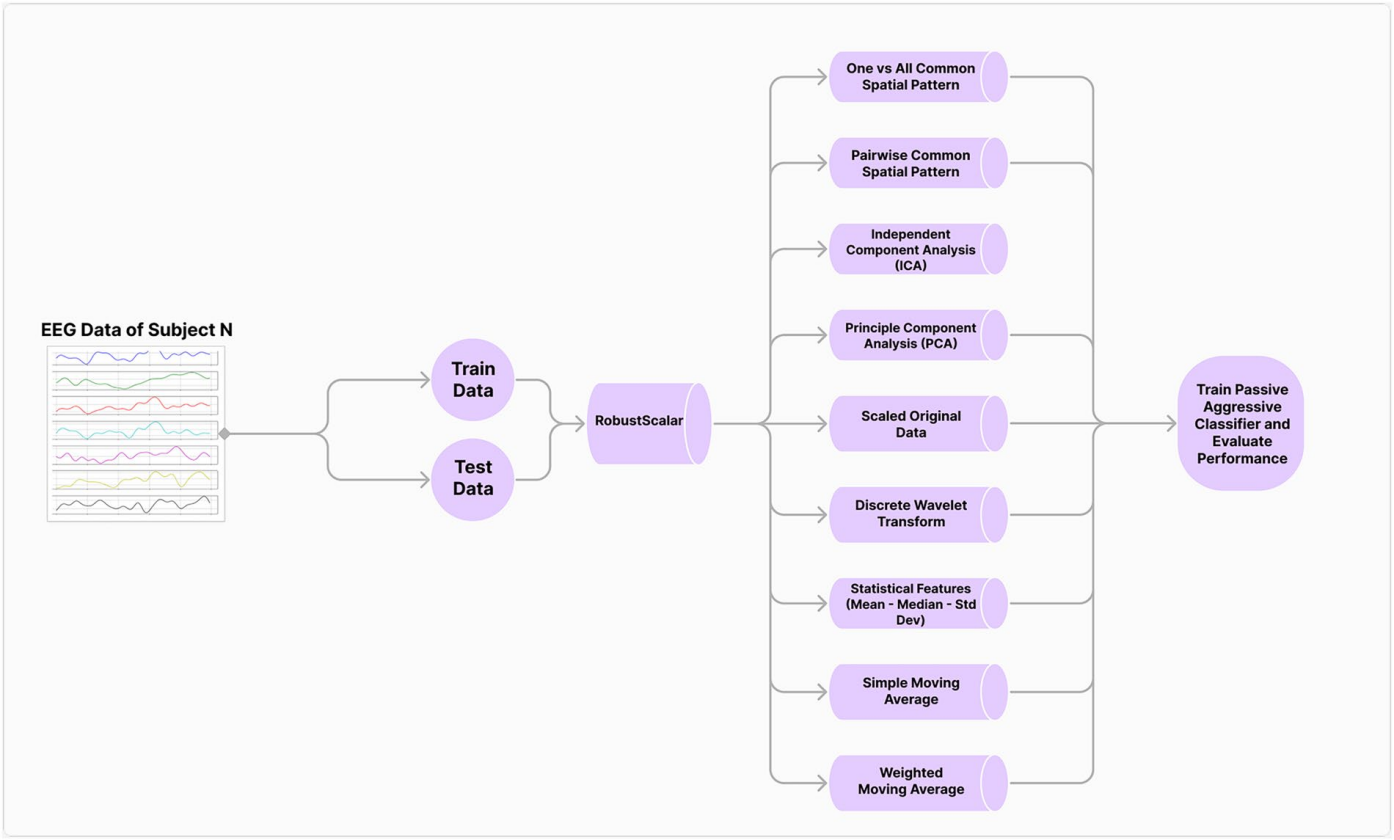
Data preprocessing and classification workflow. The dataset is split into training (80%) and testing (20%) sets. Various preprocessing methods (e.g., PCA, DWT, ICA, CSP) are applied independently before classification using multiple machine learning models. The best-performing model per subject is reported.

The evaluation of EEG data from 12 subjects involved multiple preprocessing techniques and 13 different machine learning classifiers. Initially, as depicted in Fig. 5, the data was split into training and testing datasets of 80% and 20% respectively. After the data was split, it was scaled using RobustScaler due to its reduced sensitivity to outliers. This vital characteristic makes this method stand out amongst other scaling techniques as it makes it particularly suitable for scaling EEG signals^29–31^. To ensure a reliable comparison, the original features of the data were retained alongside the transformed features to compare their accuracy.

After the previous steps were implemented, several preprocessing techniques were applied separately from each other. To capture the essential characteristics of the recorded EEG signals, statistical features, such as the mean and standard deviation were calculated. Additionally, Discrete Wavelet Transform (DWT) was used to decompose the signal into different frequency components, revealing the most important features which are not apparent in the time domain^32^. This decomposition was performed using a specified wavelet and level. Furthermore, Principal Component Analysis (PCA) was implemented to reduce the dimensionality of the data, which is necessary with the type of data being utilized in this paper, all while preserving the variance of the signals^33,34^.

After identifying the principal components using the standard implementation in Scikit-learn^35^, we experimented with various PCA configurations: retaining all components with variance greater than zero, the minimum number of components required to explain 95% of the variance, and those needed to explain 98% of the variance. This step helped remove correlated features, reduce input dimensions to allow faster training, and remove multicollinearity issues. Independent Component Analysis (ICA) was also utilized with the aim of identifying the underlying sources in the data by separating mixed signals into independent components^36^. Common Spatial Pattern (CSP) was also applied to improve the discriminative power of the EEG signals acquired by projecting the data into a new space and dimensionality to maximizes the variance between the classes^37^. In doing so, not only is the classification process enhanced and more reliable, but the brain activity observed can be understood with greater depth. Finally, Simple and Weighted Moving Averages were used to smooth the data by averaging over a window of 20 readings, which reduces the noise and highlights trends present in the data^38^. After applying each of the previously mentioned preprocessing technique separately, the results were stored in DataFrames. The DataFrames were then concatenated horizontally, forming the final training and testing features sets. This comprehensive preprocessing pipeline provided a robust feature set for subsequent classification using the Passive Aggressive Classifier^39^, as it was the best among all models tested.

**Fig. 6:**
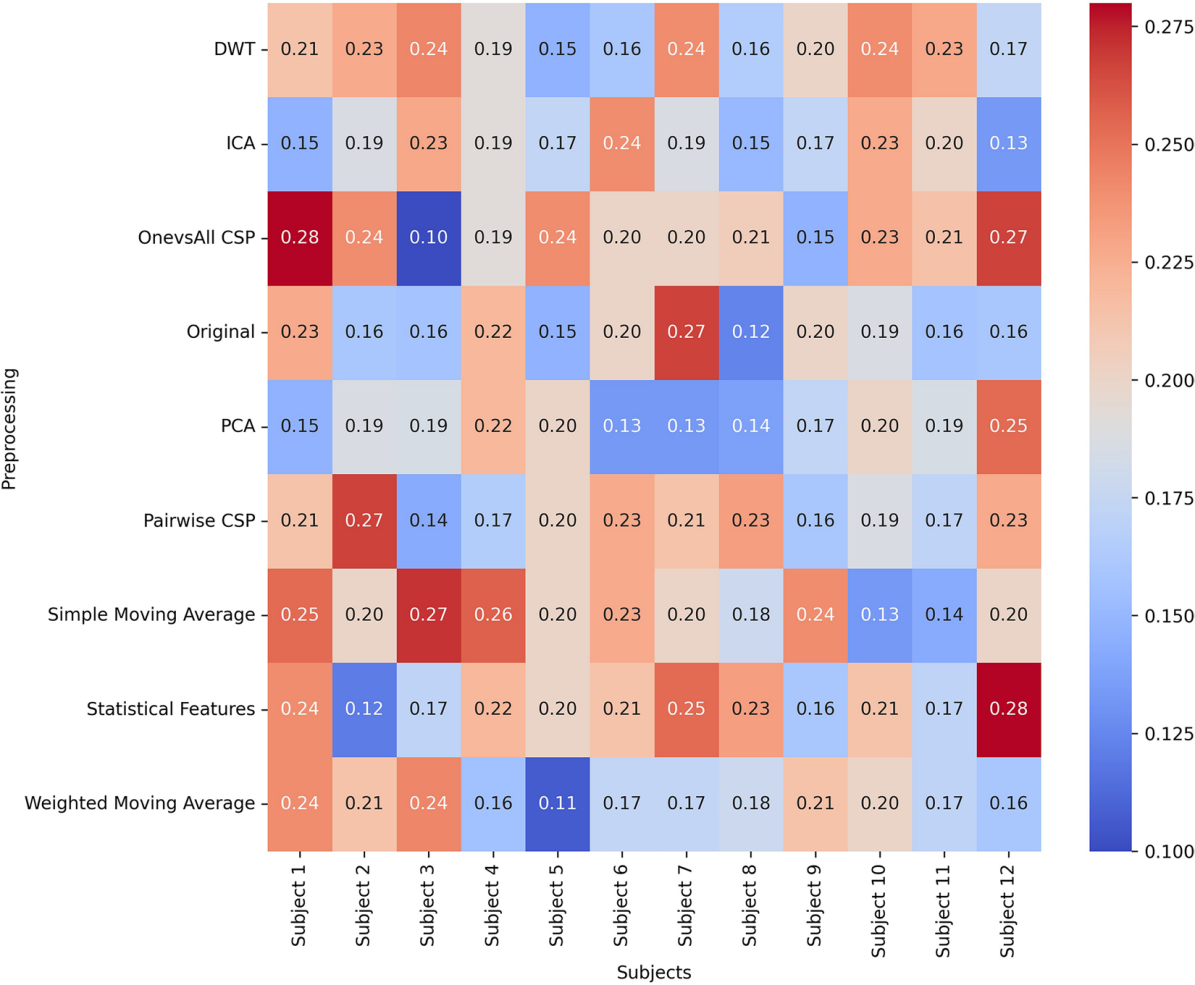
Classification accuracy for different preprocessing techniques across subjects using the Passive Aggressive Classifier. The figure shows the maximum accuracy achieved per subject using multiple pipelines. The best performance reaches 28%, which is 8% above random chance for a five-class problem.

Figure 6 illustrates the various preprocessing methodologies applied. The current evaluation focused on participant-specific performance. Group-level modeling and subject-independent classification remain key future directions for assessing the generalizability of inner speech EEG decoding. The results show that the highest accuracy achieved was 28%, which is 8% higher than the random chance for 5 classes. The results showed that the best preprocessing technique was one where the statistical features were calculated. It should be noted that while the dataset yielded accuracy levels exceeding random chance, statistical significance testing was not performed due to the limited number of participants and trials. As we continue to develop and expand the dataset, future studies will incorporate statistical validation methods such as permutation testing to support more robust performance interpretation.

### Attention monitoring

During all recording sessions, participants were accompanied by one of the authors to convey the instructions and ensure no participant mistakes were missed, such as losing focus during recording and thinking of the incorrect word. In addition, before each participant started recording, a practice trial was conducted to get the participant familiar with the setup. These practice trials are not included in the final dataset.

To ensure that the signals from inner speech had the least amount of interference from thoughts other than the word itself, three different prompt methodologies were tested. The first methodology tested was having arrow icons instead of words represent the desired prompt, as seen in Table 2. The second approach tested each participant’s focus by having them repeat the word “Up” five times consecutively before moving to the next class. This repetition was designed to reinforce the internalization of the word, thereby stabilizing the EEG signals associated with each specific inner speech task. Once that class is done, a different class is shown in the same manner. The final approach is similar to the second one, but differs in the order of the presented words. To elaborate the words in said approach were randomly shuffled rather than shown consecutively.

**Table 2:** Prompt representation: Prompt cues in the first row are the ones used in the final experiments; the second row is experimental image cues.

Rest	Up	Down	Left	Right	Select
					
					

After testing the different methodologies, the recorded data was analyzed, and it was noted that the first approach increased the noise present in the signals produced. While the consecutive sequencing approach led to participants losing their focus quickly since they could expect what the next word was, it was decided that the final approach would be used in the finalized experiment setup as it maintained the engagement of the participant during the experiment.

### Event-related potentials

**Fig. 7:**
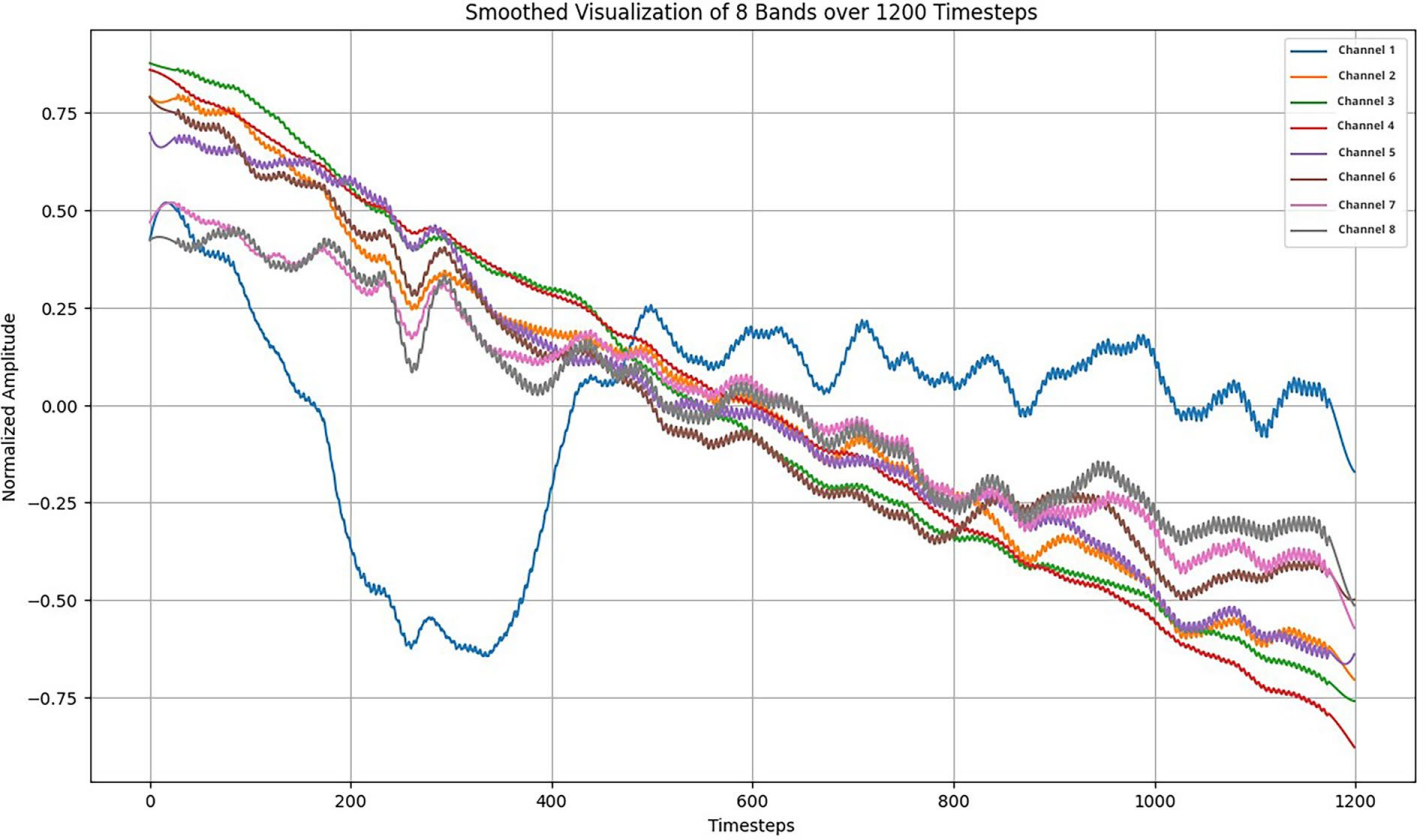
Event-Related Potentials (ERP) averaged across all participants and classes. This figure illustrates the synchronization of brain activity across the 8 EEG channels over time, centered around the onset of the inner speech cue. Notably, the first 200 timesteps (pre-cue period) show a consistent pattern across channels, suggesting a shared preparatory brain response. Following the visual cue, a distinct drop in activity is observed, accompanied by a small positive wave, likely reflecting cognitive processing of the prompt. Despite minor amplitude deviations, particularly in Channel 1, all channels maintain similar trends, supporting the temporal alignment and reliability of the captured ERP signals.

Event-Related Potentials (ERPs) are brain activities that correspond to specific stimuli^40^. In our experiments, participants were exposed to various visual cues; where these cues were expected to be accompanied by certain brain activity phenomena. These phenomena are expected to be uncorrelated with the recorded class and are expected to be clearly present across all experiments, regardless of subject. To validate these expectations, all recorded experiments were averaged across the experiments axis—originally formatted as experiments, channels, and timesteps. This process produced an average behavior profile for each channel, encompassing all subjects and classes, as illustrated in Fig. 7.

It can be seen in Fig. 7 that there are clear synchronizations between the channels at multiple sections. For instance, the behavior in the first 200 timesteps, which were the timesteps before the visual cue, shows very similar trends as the participant hears the audio cue and prepares to think of the word. The next 200 timesteps, reflecting the appearance of the cue, show a clear drop across the 8 channels, followed by a small wave bump, possibly signifying the participant’s brain activity as they recollect the shown word. The remaining timesteps begin to vary throughout the channels. It should be highlighted that certain channels in particular sections still share similarities. It can be seen that Channel 1 in particular seems to deviate notably from the rest of the channels in value without losing behavior trends. This deviation was noted and studied to assess its impact when performing aggregation but since the difference in values is not too numerically significant -still lying within the channels’ min-max range- the impact was rather minimal.

To produce the result shown in Fig. 7, the channels were centered around zero by removing the mean, the ranges were normalized by dividing by the largest absolute value, and a Savitzky-Golay filter^41^ was used to smooth the data.

### Time-frequency representation

**Fig. 8:**
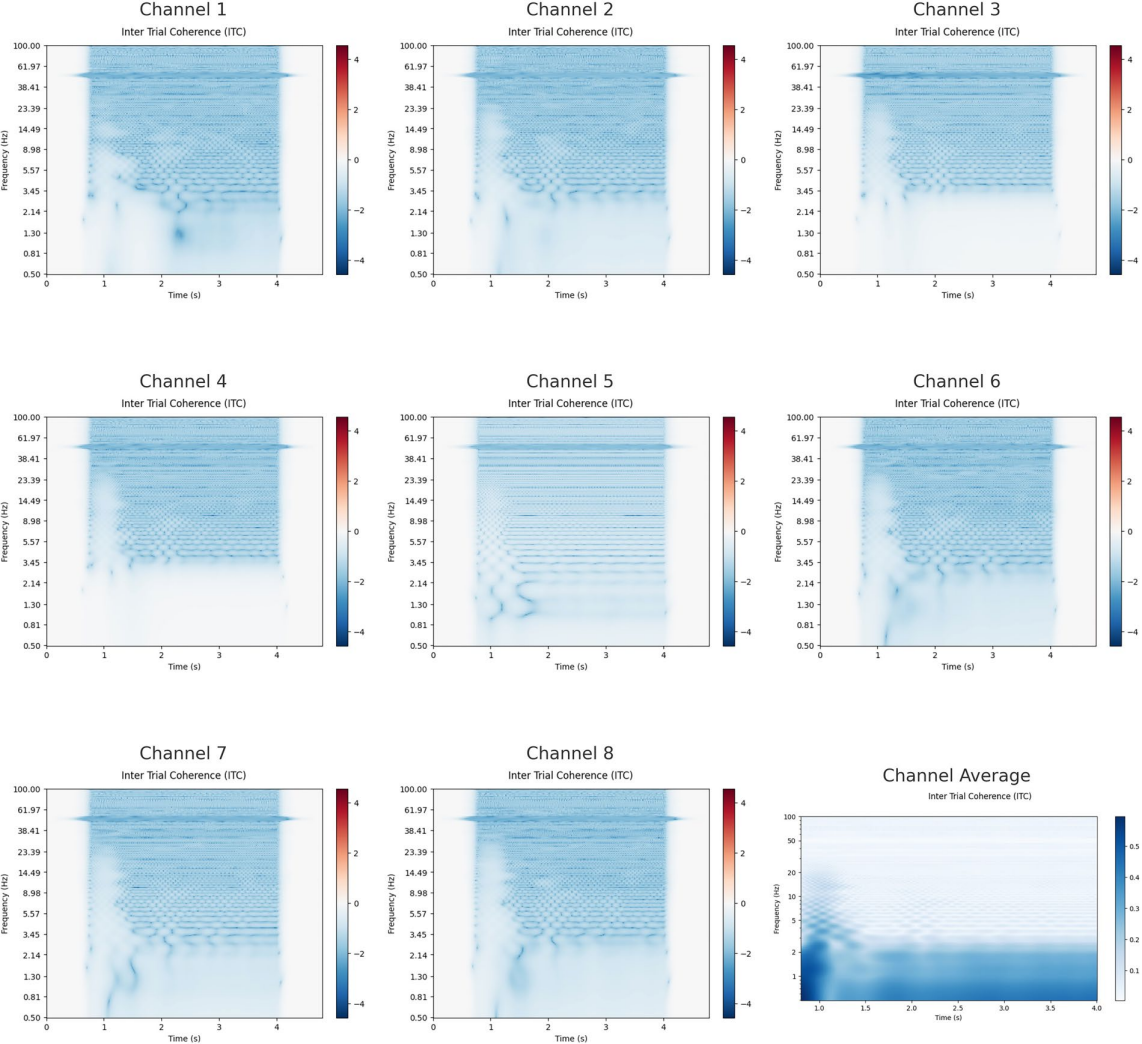
Time-frequency representation using Morlet wavelet transform. The inter-trial coherence across all trials and channels is shown, highlighting frequency bands associated with relaxation (e.g., alpha, theta) and inner speech processing. Higher coherence following the cue indicates consistent engagement across trials.

A time-frequency representation is an analysis used to identify and visualize signal characteristics across frequency and time^42^. The visual in Fig. 8 can be produced using a variety of techniques; the one utilized here is the Morlet Wavelet Transform approach^43^, which is frequently associated with EEG datasets. The MNE Python Library^28^ was used to calculate this transformation, which resulted in a complex output consisting of both real and imaginary components. The imaginary component, which represented the inter-trial coherence, is of particular interest.

The inter-trial coherence was calculated for all the trials together. The visuals for each of the 8 channels, in addition to their average, can be seen in Fig. 8. The average was cropped to only include the action interval, which is the interval immediately following the cue at approximately 0.8 seconds. The surrounding segments that occur between rests and a prompt, do not exhibit meaningful data, which is also evident in the individual channels.

In all frequency channels, especially in the lower ones like delta, theta, and alpha waves, which are linked to relaxation^44^, there is a noticeable increase in coherence among trials. This is attributed to participants being encouraged to relax to reduce signal interference. Moreover, during the second stage of the trial, coherence is notably strong across a wider range of frequencies. This consistency is expected during the period when the prompt is displayed and slightly prior, regardless of the participant.

### Power spectral density

**Fig. 9:**
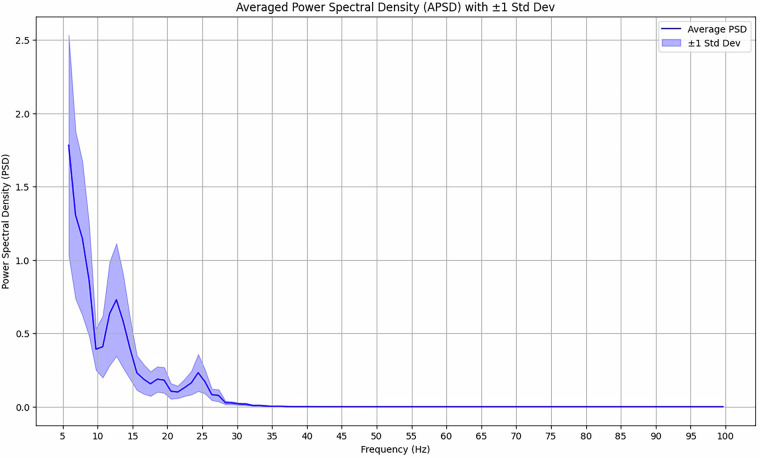
Average Power Spectral Density (PSD) across trials and channels. The figure shows averaged signal power in the 5–30 Hz range, post-filtering. Peaks are observed in the alpha (8–12 Hz) and beta (12–30 Hz) bands, supporting the dataset’s physiological relevance for cognitive and speech-related EEG studies.

The averaged power spectral density^45^ can show where most of the signal power exists across trials. It was computed across all trials from 5 Hz to 100 Hz and then averaged over the 8 channels. The visualization can be seen in Fig. 9. Shaded areas correspond to ± 1 standard deviation of all channels.

In the figure, the main two peaks seen are in the alpha band frequencies [8–12 Hz] and the beta band frequencies [12–30 Hz]^44^. These are good validations for the data, as they are expected in a dataset of this nature. It should be noted that a bandpass filter from 0.5–30 Hz (as is common in EEG datasets) and a notch filter of 50 Hz were applied for this visualization. This was done to remove the misleading power spike at 50 Hz, which originates from power line interference^46^, as well as remove higher frequency powers, which are likely noise in the context of EEG signals.

## Limitations & Remarks

This work introduces a new inner speech EEG dataset to the scientific community, designed to support advancements in EEG modeling. By incorporating Arabic classes, this dataset not only promotes the adoption of EEG technology in Arabic-speaking countries and the MENA region but also aims to advance global research in this field. Recorded using a cost-effective 8-channel EEG headset by native Arabic speakers, it enhances the representation of Arabic data within EEG research. The introduction of this dataset is a step towards improving daily communication for individuals with communication impairments, contributing significantly to technological inclusivity and accessibility.

It should be mentioned that, like any other EEG or brain-related dataset, despite the fact that participants were clearly instructed, encouraged, and assisted to think of the prompted words on the screen, humans can get distracted easily. Additionally, the incredible complexity of the brain further adds to the difficulty of capturing the waves that truly correlate with inner speech. In other words, there is no method that will guarantee that all the participants were genuinely thinking of the correct word at the correct time or that their interpretation of the instructions does not differ from one individual to another. However, it is hoped that across a large enough number of trials, some common element can be discovered that can be used for effective classification.

While this research aims to demonstrates the feasibility of inner speech decoding using a low-density EEG setup, it is important to note that multiple aspects remain to be investigated. For instance, the statistical significance testing was not performed due to dataset size constraints. Additionally, the evaluation was conducted using participant-specific pipelines, and generalizability to unseen subjects was not assessed. Accordingly, these are important directions for future work, where we aim to expand the dataset, incorporate group-level modeling, and conduct statistical validation to support broader applicability.

Inner speech, despite the limited evidence that supports and validates its effectiveness as a classifiable command method for BCI, remains the most promising option for real-life extensible applications. The reliance on gestures or muscular movement as well as external stimulants would void the benefits of the use of any BCI system in real-life situations. Hence, this dataset is another piece added to the puzzle of deciphering inner speech in the hopes of creating a viable evasive EEG solution for BCI applications.

## Usage Notes

All codes used in this study are available at the GitHub repository linked in the section below. All the required packages are provided in the “requirements.txt” file. For analysis purposes the main file to use is the “dataloader.py” which has a function “LoadAllSubjects” that downloads the data from OpenNeuro^26^ repository, applies the preprocessing mentioned in the paper and returns a Pandas^24^ DataFrame with the data. For the data collection code, it will be available in the “Recording” folder. The main file is “PromptViewer.py” which should be run directly. It’s recommended to follow^18^ for more instructions on using the Unicorn Headset utilised in this paper.

## Data Availability

All codes used in this paper, such as the code for the data collection program used to collect and annotate the data, as well as the preprocessing code is available in (https://github.com/Eslam21/ArEEG-an-Open-Access-Arabic-Inner-Speech-EEG-Dataset). Furthermore, the link to the dataset itself can be found in the GitHub repository mentioned through the OpenNeuro database^26^. Furthermore, while the preprocessing code is left as it was used in this paper, there is room for future researchers to change the parameters and preprocessing done according to their objective and analyse how said changes affect the signals.
